# Economic Assessment of Bioethanol Recovery Using Membrane Distillation for Food Waste Fermentation

**DOI:** 10.3390/bioengineering7010015

**Published:** 2020-02-11

**Authors:** Noor Intan Shafinas Muhammad, Kurt A. Rosentrater

**Affiliations:** 1 Agricultural & Biosystems Engineering Department, Iowa State University, Elings Hall, Ames, IA 50011, USA; shafinas@ump.edu.my; 2 Faculty of Chemical and Process Engineering Technology, University Malaysia Pahang, Lebuhraya Tun Razak, Kuantan 26300, Malaysia

**Keywords:** food waste fermentation, ethanol, membrane distillation, column distillation, costs

## Abstract

Ethanol is a material that has a high demand from different industries such as fuel, beverages, and other industrial applications. Commonly, ethanol has been produced from yeast fermentation using sugar crops as a feedstock. However, food waste (FW) was found to be one of the promising resources to produce ethanol because it contained a higher amount of glucose. Generally, column distillation has been used to separate ethanol from the fermentation broth, but this operation is considered an energy-intensive process. On the contrary, membrane distillation is expected to be more practical and cost-effective because of its lower energy requirement. Therefore, this study aims to make a comparison of economic performance on FW fermentation with membrane distillation and a conventional distillation system using techno-economy analysis (TEA) method. A commercial-scale FW fermentation plant was modeled using SuperPro Designer V9.0 Modeling. Discounted cash flow analysis was employed to determine ethanol minimum selling price (MSP) for both distillation systems at 10% of the internal rate of return. Results from this analysis showed that membrane distillation has a higher MSP than a conventional process, $6.24 and $2.41 per gallon ($1.65 and $0.64 per liter) respectively. Hence, this study found that membrane distillation is not economical to be implemented in commercial-scale ethanol production.

## 1. Introduction

Food waste (FW) is considered a growing problem in the world. This problem occurs throughout the food supply chain, from production to human consumption. In 2014, the United States generated 38 million tons of food waste yearly [1]. There are various factors in FW generation, such as spillage, inefficient storage facilities, spoilage, environmental change, and human behavior. Although these causes can be reduced by engineering control, behavior and attitude are the most challenging factors to manage [2].

FW is expected to rise every year due to population, economic growth, and unhealthy lifestyles [3]. Greenhouse gas (GHG) emissions, climate change, water footprint, sanitation, health, and ecological and economic effects are caused by food waste [4,5]. Landfilling is a convenient option as an FW disposal method. However, this method is not sustainable due to land limitation, especially in an urban area [6]. Thus, it is essential to find a new strategy for FW disposal that at the same time could reduce the environmental burden while producing a high-value product.

Two widely used methods for food waste conversion are biochemical (e.g., fermentation and anaerobic digestion) and thermochemical (e.g., incineration, pyrolysis, and gasification). However, according to Pham et al. [7], incineration, pyrolysis, and gasification methods are not suitable because of the higher moisture content of FW and require higher energy to process. As for anaerobic digestion, higher capital cost and adverse environmental impact make this method not favorable. Hence, the fermentation method is considered an effective method because of glucose content which is suitable for *Saccharomyces cerevisiae* to ferment FW into ethanol. Ethanol is an organic compound that has a demand in different industries such as transportation fuel, cosmetics, pharmaceuticals, and household products.

A study performed by Muhammad [8] found that FW fermentation without enzymes and with a two-step distillation system is more economical in producing ethanol as the main product. From the results, this process has the lowest ethanol minimum selling price (MSP), which is $2.41 per gallon ($0.64 per liter). In this process, two-column distillation was used to separate ethanol from the fermentation broth. This separation process is widely implemented in the ethanol industry.

However, the distillation column method is considered as an energy-intensive process, which could increase the cost [9]. At least 40% of the total energy consumption in ethanol production is coming from the distillation process [10]. Several methods are recommended to substitute the distillation process, such as reverse osmosis and membrane distillations [11]. The membrane provides an alternative option, either using hydrophobic or hydrophilic membranes to separate ethanol from the fermentation broth. Membrane distillation (MD) is one of the emerging technologies that has gained more attention from researchers and industries. In this process, the separation process can occur below the average boiling point of the solution. Moreover, the membrane performance varies based on membrane selectivity, operational conditions, and types and size of polymers [12]. Therefore, membrane distillation is considered more efficient, easy to operate, and with a low energy requirement [11,13,14,15,16]. There are several studies that have been done by previous researchers regarding membrane distillation for ethanol production from different feedstock. The results show that MD is one of the alternative ways to separate ethanol from the conversion process. Hence, MD it is expected to improve the process economy.

**Figure 1:**
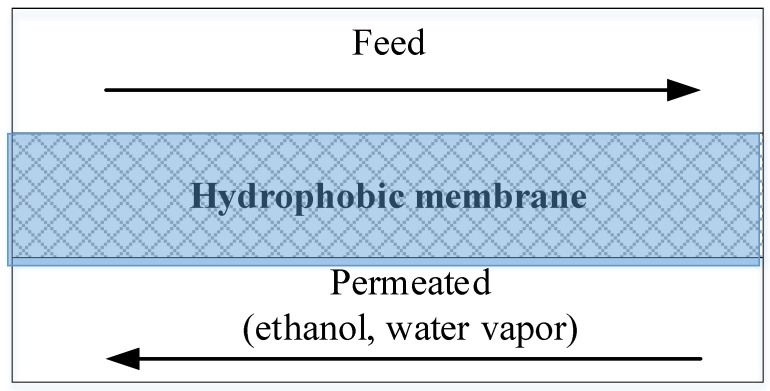
Schematic diagram of the membrane distillation process.

In this study, a hydrophobic porous membrane was used for the MD method. The driving force in this system is maintained by the differential pressure on both sides of the layer due to the temperature difference. The general schematic diagram of membrane operation is shown in Figure 1. The feed stream temperature is suggested to be higher so that the desired components can diffuse through the membrane. According to Baeyens et al. [15], the permeate flux increases significantly with increasing feed temperature in a range from 37 to 61 °C. Similarly, findings from Banat and Simadl [17], show that in the range of 40–70 °C feed temperature, 2–3.5 of ethanol selectivity can be achieved.

Therefore, the main focus of this study was to make a comparison of the economic performance of FW fermentation with membrane distillation and conventional distillation column in the ethanol separation process. Techno-economic analysis (TEA) will be used to estimate the ethanol minimum selling price (MSP) per gallon for both separation processes. The economic performance for the distillation column was used from a study reported by Muhammad [8].

## 2. Methodology

### 2.1. Process Modeling

**Figure 2:**
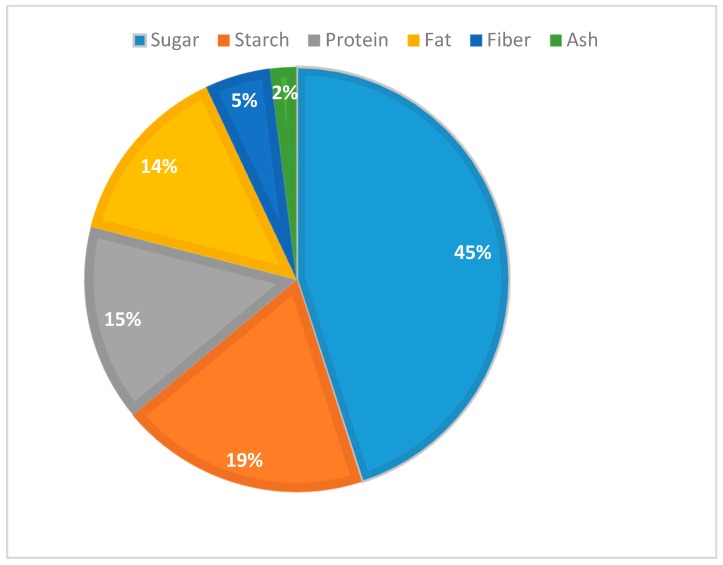
Average value of food waste (FW) composition (% w/w wet basis) [8].

The food waste composition is illustrated in Figure 2, and the fermentation process was modeled in an open anaerobic condition with ethanol yield as 2.2% (w/w) wet basis without any enzymes. The FW conversion and ethanol yield were taken from the experimental study done by Muhammad [8]. MD was designed as the ethanol separation process using SuperPro Designer V9.0 for evaluating the plant performance on a commercial scale. All necessary unit operations for all relevant processing steps were modeled.

The daily plant feedstock was assumed to be 2000 Mg/day at no cost. The moisture content of solid waste from this separation process was maintained at 40% by weight to control the microbial activity.

MD was used in this processing plant because this process was expected to have a low-temperature heat requirement, which could be economical in cases when waste is used. In this simulation, the fermentation broth was be heated to 37 °C for obtaining a permeate flux at 0.32 g/m^2^ s [15].

**Figure 3:**
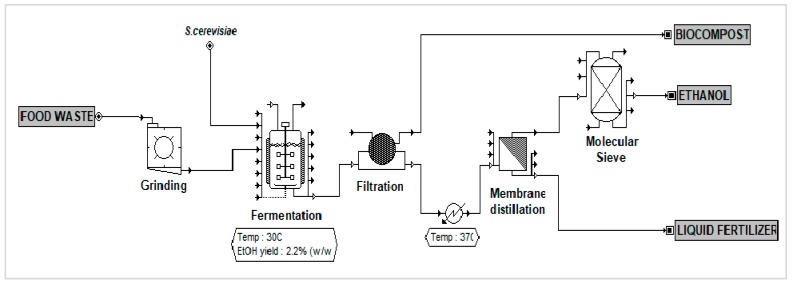
Process flow diagram of FW fermentation with membrane distillation separation process.

**Figure 4:**
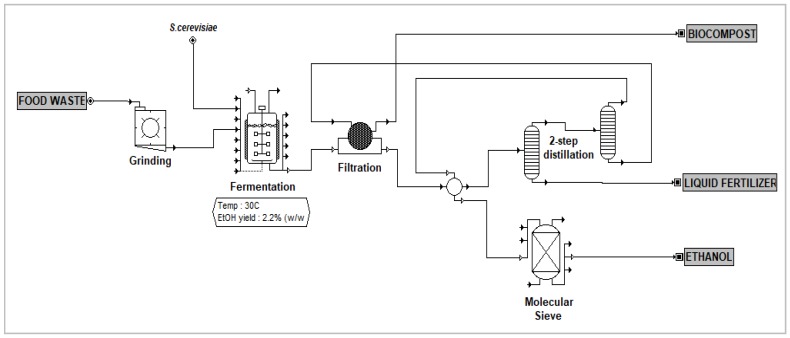
Process flow diagram for FW fermentation process without enzyme and two-step distillation system.

The size and quantity of equipment, utilities and energy consumptions, transportation cost, labor, and raw material needed were determined by mass and energy balance from the simulation. The plant was expected to operate at least 7900 hours per year. The overall process flow using MD as downstream processing is illustrated in Figure 3. For comparison, Figure 4 shows the process flow diagram for FW fermentation without enzymes and two-step distillation as ethanol separation.

### 2.2. Techno-Economic Assumptions

In this study, a list of assumptions was made for the operation process and economic evaluation. The cost of equipment purchased was taken from developed models in SuperPro Designer V9.0 and indexed to 2018 dollars. The methods to calculate the project investment expenditure were adopted from Peters et al. [18], which are commonly accurate within 30%. In addition, a 3.02 installation factor was used because it is a common assumption factor for biorenewable facilities plant [9]. A discounted cash flow analysis spreadsheet was used to estimate the MSP value ($/gal) with a zero net present value (NPV) and a predetermined internal rate of return. The main assumptions made in this study for both processing plants are listed below.

- Plant capacity: 2000 Mg/day (tonne/day)
- Plant feedstock: FW with 78% moisture content
- Plant distance: 12 miles (19.3 km) radius [19]
- Plant life: 20 years
- The internal rate of return (IRR): 10% [20]
- Equity financed: 100%
- Plant depreciation: 7 years with 200% double declining balance (DDB)
- Contingency factor: 20% from total installed equipment and indirect cost
- Construction period: 2.5 years with total capital investment spent with 8%, 60%, and 32% for first, second, and third year, respectively.
- Startup period: 6 months, considering 50% of revenues, 75% variable cost, and 100% fixed expenses will be achieved.

As mentioned previously, biocompost was be considered as co-product and can be sold with organic fertilizer in the agricultural market to optimize the operational profit. The selling price of bio-compost was assumed to be 8 ¢/lb (17.64 ¢/kg) based on the average organic fertilizer price in Iowa [21].

Economies of scale will be performed in this study to evaluate the reduction of the product value of increasing daily feedstock volume from 10 to 5000 Mg. From this analysis, the range of optimum feedstock value with the lower MSP value will be estimated for future study.

### 2.3. Sensitivity Analysis

Further analysis was required to identify the parameter with the most significant impact on MSP value. A sensitivity analysis is a method by modifying one parameter value while maintaining others. Table 1 shows the sensitivity analysis parameters selected for this analysis. These parameters are identified as a powerful impact on plant economic performance.

## 3. Results and Discussions

### 3.1. Economic Analysis

This plant was designed to have a feedstock capacity at 2000 Mg/day of FW. The mass and energy balance was obtained from the simulation result. From the discounted cash flow analysis, the MSP value was estimated to $6.24 per gallon ($1.65 per liter) with yielding an NPV of zero and 10% IRR.

**Figure 5:**
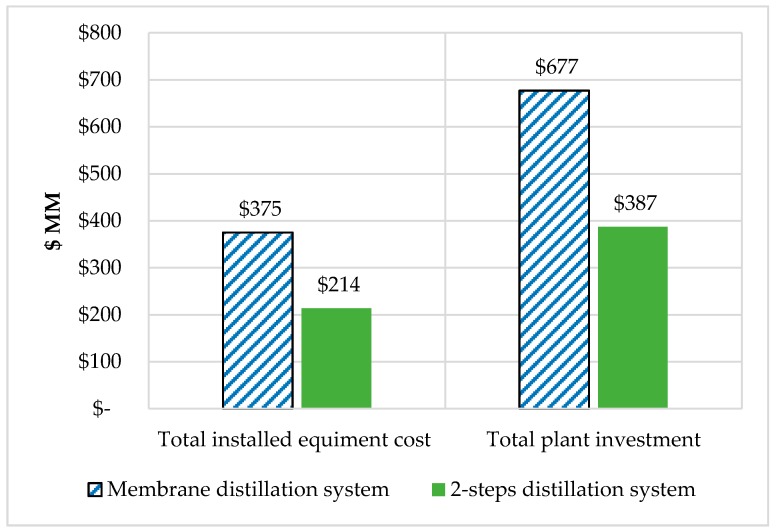
Comparison of capital investment of FW fermentation with membrane distillation and two-step distillation system for the separation process.

This plant has a value for total installed equipment cost (TIEC) and total project investment (TPI) of $375 MM and $677 MM, respectively. In addition to that, annual utility cost ($/year) and labor cost ($/year) demand amount to $26 MM and $1.1 MM, correspondingly. From a study done by Muhammad [8], FW fermentation without enzymes and two distillation column have the best economic performance. If compared with this system, the distillation column was better than membrane distillation regarding capital investment as illustrated in Figure 5. Similarly, Peiter et al. [22] found that membrane distillation has a higher investment than distillations, due to the fact that the membrane has a limited lifespan and expenses that make it not practical to be used on a commercial scale.

Moreover, more units of membrane distillation were required due to the fouling factor. The accumulation of deposit on the surface clogs the pore and reduces the permeability. Thus, it could reduce the separation process efficiency.

Higher energy consumption is the main reason that a distillation column is not favorable. In this analysis, energy was counted in utility cost, which was considered as standard electric power, steam, water, cooling, and chilled water. The price for each unit of utilities is shown in Table 2. Price for electricity and water are taken from the U.S. Energy Information Administration (EIA) [23], while that for steam, cooling, and chilled water are taken from the default setting in the SuperPro Designer V9.0 software.

**Figure 6:**
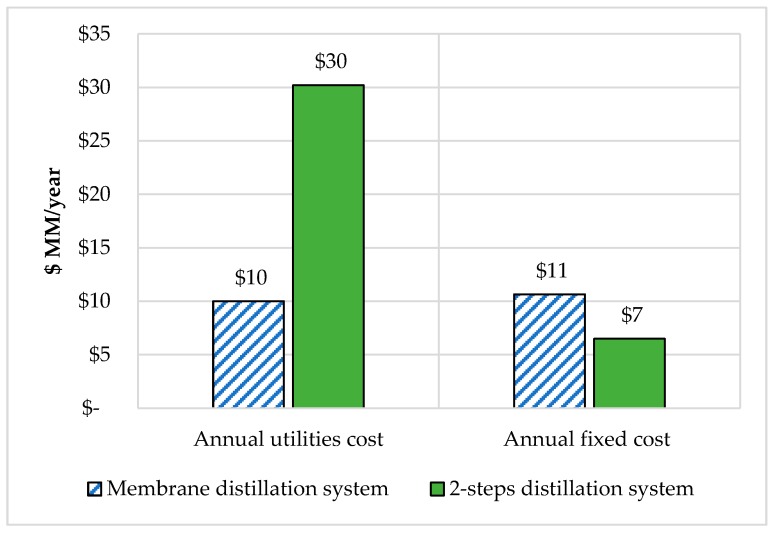
Comparison of variable and fixed cost of FW fermentation with a two-step distillation system and membrane distillation separation process.

Results from the economic analysis showed that membrane distillation has the lowest utility price compared to the distillation column. This finding supports the idea that the distillation column requires more energy compared to the membrane. However, annually, fixed-cost membrane distillation cost 36% more than the distillation column. As mentioned above, the membrane needs more units and thus will increase the labor, maintenance, and operating cost. The comparison of both systems is clearly shown in Figure 6.

**Figure 7:**
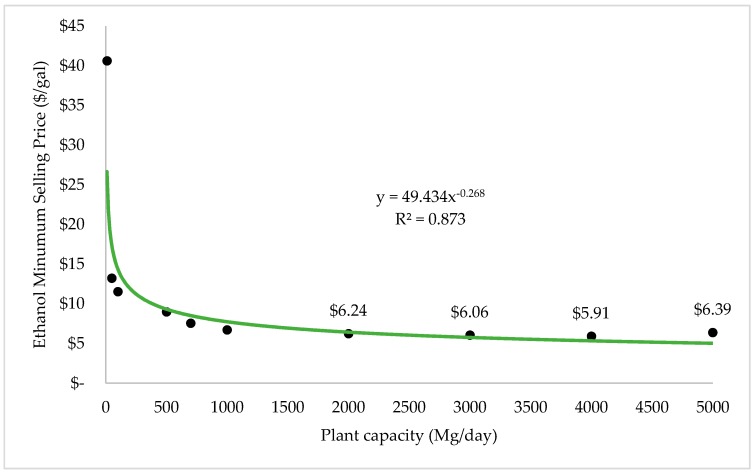
Economies of scale of FW fermentation with membrane distillation separation process.

Economies of scale for the membrane distillation study are represented in Figure 7. From the graph, there was a power relationship of −0.268 between MSP and feedstock size. It also shows that with the feedstock rate varying between 10 and 5000 Mg per day, the MSP of ethanol ranges from $40.62 to $6.39 per gallon ($10.73 to $1.69 per liter). It clearly shows that the diseconomies of scale happen when the plant capacity increases to 3000 Mg per day. Thus, the size of the feedstock input value should not be larger than 3000 Mg daily to make the project economically feasible.

### 3.2. Sensitivity Analysis

**Figure 8:**
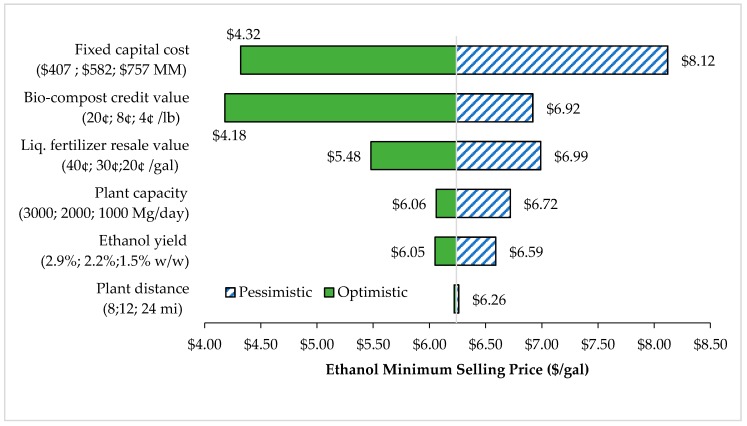
Sensitivity analysis of FW fermentation with membrane distillation separation process. (Optimistic is the best-case scenario simulation, pessimistic is the worst-case scenario simulation).

Figure 8 shows the sensitivity analysis for the membrane distillation separation process. The tornado chart indicates that fixed capital cost was the most influential parameter in estimating the MSP value. The increasing amount of capital cost from $407 MM to $757 MM elevates the MSP value by 88%. As discussed previously, membrane distillation has a higher capital and operational cost because more separation units were required with the higher cost of a membrane.

## 4. Conclusions

The techno-economic analysis evaluates the production cost of FW fermentation with two separation processes: membrane and distillation system. Based on the mass balance, both of the methods could potentially recover most of the ethanol with a further purification system. The membrane had less energy demand, but it had higher capital and operational costs. The result from a discounted cash flow analysis showed that the MSP for membrane distillation was higher over the conventional distillation system, with estimated value are $6.24/gal and $2.41/gal ($1.65/liter and $0.64/liter), respectively. The negative economic impact of membrane distillation was one of the most challenging factors and makes it not favorable.

Similarly, the selectivity membrane materials are expensive and have a shorter lifespan and were not considered in this study. Thus, it could increase the capital and maintenance cost. Overall, the total plant investment and annual fixed cost were the factors driving the increase in product cost.

Sensitivity analysis was the method to find the parameters that strongly impact the estimation of MSP value. The variability of capital cost at ± 30% would result in MSP in a range of $4.32 to $8.12 per gallon ($1.14 to $2.15 per liter).

Findings from this study could provide a view on possible solutions to convert FW into value-added products. Although anaerobic digestion could be a selected method to deal with FW, it will also entail some disadvantages to the environment. FW fermentation is a potential method for solving the problems of food waste management. The economic analysis revealed that membrane distillation is not cost effective for industrial bioethanol production, although it has been considered a more environmentally friendly process. Further work on new methods which are economically viable needs to be developed. For example, a modification of product harvesting mode to enhance the separation process may be one of these possibilities.

## Figures and Tables

**Table 1:** Sensitivity analysis parameters for FW fermentation with the membrane distillation separation process.

Parameters	Optimistic	Base Case	Pessimistic
Plant distance—miles radius (km radius)	8 (12.9)	12 (19.3)	24 (38.6)
Bio-compost resale value—¢/lb (¢/kg)	20 (44.1)	8 (17.6)	4 (8.8)
Plant Capacity—Mg/day	1000	2000	3000
Liq. Fertilizer resale value—¢/gal (¢/L)	40 (10.6)	30 (7.9)	20 (5.3)
ethanol yield (% w/w) wet basis	2.9	2.2	1.5
Fix capital cost ($MM)	407	585	757

**Table 2:** Utility prices [19].

Utility Component.	Prices
Electricity (¢/Kwh)	5.5
Water (¢/gal) (¢/liter)	0.35 (0.09)
Steam ($/Mg)	12.00
Cooling water ($/Mg)	0.05
Chilled water ($/Mg)	0.40
